# Waldenström Macroglobulinemia in Hepatitis C: Case Report and Review of the Current Literature

**DOI:** 10.1155/2014/165670

**Published:** 2014-08-27

**Authors:** Ryan Nipp, Aaron Mitchell, Allyson Pishko, Ara Metjian

**Affiliations:** ^1^Dana-Farber Cancer Institute, 450 Brookline Avenue, Boston, MA 02215, USA; ^2^Department of Medicine, Duke University Medical Center, 2301 Erwin Road, P.O. Box 3422, Durham, NC 27710, USA; ^3^Internal Medicine Residency Office, Duke University Hospital, 2301 Erwin Road, Durham, NC 27710, USA

## Abstract

*Background*. Recent literature has associated hepatitis C virus with the development of non-Hodgkin lymphoma. Hepatitis C virus infection appears to promote lymphoproliferation, providing a plausible mechanism for a causative association; however, despite prior reports of patients with comorbid hepatitis C infection and Waldenström macroglobulinemia, the literature is in disagreement regarding whether there exists an association between these two conditions. *Case Presentation*. This case report describes a 57-year-old African-American male with chronic hepatitis C infection and cryoglobulinemia who presented with several episodes of transient confusion and paralysis and was found to have symptomatic hyperviscosity. The recognition of his condition was facilitated by characteristic findings on ophthalmologic examination. He was subsequently diagnosed with Waldenström macroglobulinemia on bone marrow biopsy. *Conclusions*. An up to date, comprehensive review of the literature suggests an association between hepatitis C and Waldenström macroglobulinemia. Data on optimal treatment of patients with comorbid hepatitis C infection and Waldenström macroglobulinemia is limited. We have provided a comprehensive review of previously explored treatment options to guide management of other similar patients. Our patient has since been treated with repeated plasmapheresis with a plan to pursue antiviral therapy.

## 1. Background

Waldenström macroglobulinemia (WM) is a lymphoproliferative B-cell disorder characterized by both an immunoglobulin (Ig) M monoclonal protein in the serum and lymphoplasmacytic cells in the bone marrow (BM). Like WM, cryoglobulinemia is associated with chronic lymphoproliferation and paraprotein production. Cryoglobulinemia is common in WM (8–18% of patients) as well as in hepatitis C virus (HCV) infection. The mutual association of both WM and HCV with cryoglobulinemia has led to speculation that HCV infection may play a causative role in WM and/or other non-Hodgkin lymphomas (NHL) [1].

**Figure 1:**
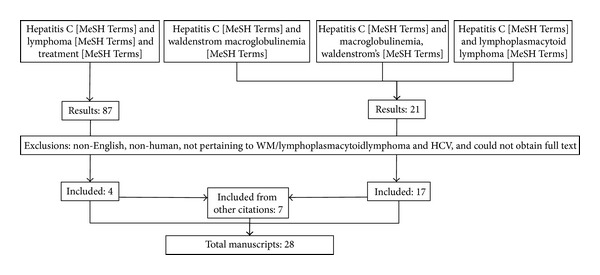
CONSORT diagram. Articles were selected for review using the following queries.

Plausible mechanisms for a role of chronic HCV infection in the development of WM include both cytokine-mediated and direct viral stimulation of B-lymphocytes, triggering clonal proliferation and progression to lymphoid malignancy in some patients. However, there is disagreement in the literature as to whether this hypothetical association between HCV and WM exists in practice [2]. To fully assess the question of a possible role of HCV in the pathogenesis of WM, we conducted a comprehensive literature search for articles pertaining to both HCV and WM or treatment of NHL (Figure 1), yielding a total of 28 articles (Table 1).

Some epidemiologic studies have found evidence supporting an association between HCV and NHL. A meta-analysis found the prevalence of HCV in patients with B-cell NHL to be approximately 15%, markedly higher than in both the general population (1.5%) and patients with other hematologic malignancies (2.9%) [3]. An Italian group found a greater prevalence of HCV infection among patients with NHL (37.1%) than among age- and sex-matched controls (8.6–9.7%) [4].

In addition to an association with NHL, other data support an association between HCV and WM in particular. Numerous studies suggest that WM is the most frequent NHL subtype among HCV-related NHLs, with HCV infection found in 26–49% of WM cases [5]. A 1993 study found that 6/6 patients with WM were infected with HCV [6]. A United States Veteran's Affairs health facilities study found that HCV-infected patients had an almost 3-fold increased risk of WM (HR 2.76 [95% CI 2.01–3.79]) [7]. Another large population study found that HCV infection was associated with an increased risk of NHL overall (OR 2.2; CI 0.9–5.3) and of WM specifically (OR 5.2; CI 1.0–26.4) [8]. A study of Japanese patients revealed that 16% of patients with known B-cell malignancy tested positive for HCV-RNA, while no patients were HCV-positive in the control group [9]. Of the 4 patients with WM, 1 tested positive for HCV [9]. Another study of HCV infection in cryoglobulinemic and noncryoglobulinemic B-cell NHL found that 16/17 WM patients were HCV-Ab positive (RR = 32.88, CI 24.42–39.43) [10].

Despite these findings, other data argue against an association between HCV and WM [2, 3, 11–13]. One survey of WM patients revealed a prevalence of HCV (4.4%) comparable to the population average [11]. Another study was unable to demonstrate a significant association between the development of lymphoma and HCV infection (OR 2.02; CI 0.34–11) [3]. A cross-sectional investigation of 100 randomly selected patients with WM found a 0% rate of HCV positivity [2]. A retrospective cohort of 95 patients with B-cell lymphoproliferative malignancies found that none of these patients had been HCV-positive at the time of a remote serum preservation [12].

Here, we report a case of a man with known chronic HCV infection who presented with cryoglobulinemia and was found to have WM. Furthermore, we describe our chosen treatment course and provide a review of the literature on available treatment strategies for patients in which these conditions are comorbid.

## 2. Case

A 57-year-old African-American man presented to the emergency department with acute loss of consciousness.

Medical history included chronic, untreated HCV without known cirrhosis, hypertension, cocaine use, type 2 diabetes mellitus complicated by retinopathy, and right knee replacement for osteoarthritis. Over the past year, he had experienced episodic melena which did not fully resolve after photocoagulation of gastric ulcers, resulting in anemia and necessitating several blood transfusions during this time.

In the several months prior to presentation, he experienced increasing malaise and a gradual decline in his visual acuity. In this context, he suffered four episodes of transient extremity paralysis, confusion, and gait instability, receiving medical attention after each. He initially received the diagnosis of transient ischemic attacks and was treated with aspirin-dipyridamole, which was later discontinued due to new onset of recurrent epistaxis.

**Figure 2:**
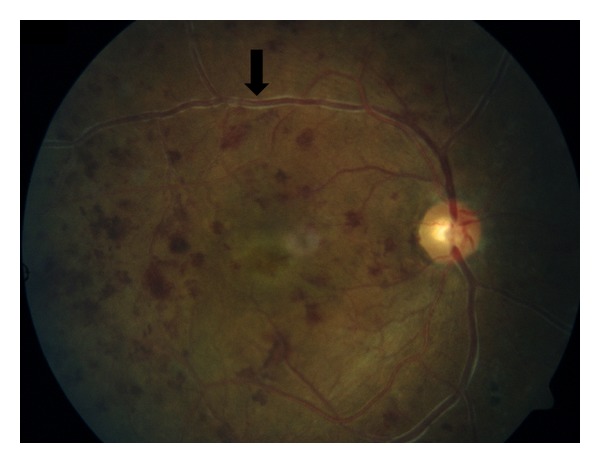
Ophthalmologic findings in the presented case. Optic disk of the left eye is shown. Perivenous sheathing is indicated (black arrow).

He experienced the fifth episode of confusion and paralysis while being at a restaurant with his daughter, this time eventually collapsing and becoming nonresponsive. By the time of ED arrival, he had returned to baseline mental status and vital signs were within normal limits. Physical exam was remarkable for visual acuity of 20/50 in the right eye and 20/70 in the left, and a fundoscopic exam revealing multiple, bilateral dot-blot hemorrhages, perivenous sheathing, and macular edema, consistent with central retinal vein occlusion (Figure 2).

Initially, laboratory data were unavailable, as the patient's high blood viscosity prevented testing. Cryoglobulin screen was positive, and cryocrit was 35 mm (reference range 0–2 mm). On hospital day 2, he began to have epistaxis that was refractory to management by otolaryngology consultation, and the following day he was treated with plasmapheresis. Epistaxis resolved, and he reported feeling “better than I've felt in years.”

Further testing performed after initial plasmapheresis showed a serum relative viscosity of 3.3 (normal saline reference; normal serum range 1.6–1.9). Hemoglobin was 8.2 g/dL, and leukocytes were 4600/*μ*L. HCV titer was 96,800 copies/mL. Serum and urine protein electrophoresis with immunofixation showed a monoclonal IgM kappa. Bone marrow biopsy showed monoclonal, kappa light chain-restricted populations of plasmacytic B lymphocytes and plasma cells, diagnostic of WM.

The patient's course was later complicated by right knee prosthetic joint infection. He required multiple courses of antibiotics and his need for total knee arthroplasty reimplantation delayed definitive treatment with antiviral therapies. Plasmapheresis, performed every 2 weeks, was used successfully as a temporizing measure during this time. Seventeen months after initial presentation, he was started on simeprevir, pegylated interferon, and ribavirin for treatment of HCV.

## 3. Conclusions

While the debate over the association and possible etiologic role of HCV in WM continues, the literature on treating patients with WM and coexisting HCV infection is even sparser, mostly from case reports. In our literature review, we found a paucity of data on optimal treatment strategies for patients with both HCV and WM or other NHLs (Table 1). In particular, there are no US FDA-approved therapeutic agents for WM despite the growing advances in understanding WM [14]. Treatment options for patients with comorbid HCV and NHL include antivirals targeting the HCV or chemotherapeutics targeting the lymphoma. A literature review from 1997 postulated that interferon (IFN) treatment might be efficacious for HCV-related NHL, describing the regression of monoclonal B-cell expansion in those patients who cleared HCV following IFN treatment [15]. Another 2012 systematic review of HCV-associated B-cell lymphoma concluded that anti-HCV treatment with IFN and ribavirin is appropriate in select cases of B-cell NHL, also noting that the lymphoma regression seen after IFN-based treatment strongly supports a causative role for HCV in NHL [16]. Alternatively, regarding treatment strategies targeting the lymphoma, a review concluded that chemotherapy is generally well tolerated in lymphoma patients with comorbid HCV infection; even bone marrow transplantation has been used successfully in this setting [5].

There exist few case reports outlining the application of these treatment strategies to WM in particular. Treatment with IFN-alpha and ribavirin was reported in a patient with HCV-associated cryoglobulinemia and WM [17]. After 9 months of antiviral therapy, the patient's HCV titers became undetectable, and bone marrow biopsy revealed regression of lymphoid infiltration. Eventually, a liver transplant was performed and the patient remained asymptomatic [17]. Another case report of comorbid HCV and WM described a combined cytotoxic and antiviral strategy with cyclophosphamide, prednisone, pegylated IFN, and alpha-2b treatments. Four months after cytotoxic and antiviral treatments ceased, the patient developed nephritic syndrome and died with sepsis [18]. Another patient with known HCV cirrhosis and recently diagnosed WM developed nephrotic syndrome; he was treated with repeated plasmapheresis, but the nephrosis progressed and he died 15 months after diagnosis [19]. Also reported was a patient with comorbid HCV and WM managed with repeated administration of melphalan, prednisolone, and vincristine over 4 years until developing and succumbing to hepatocellular carcinoma [20].

We have described the presentation of an African-American man with chronic HCV infection who later developed cryoglobulinemia and was diagnosed with WM. He has been treated with weekly plasma exchange therapy while awaiting antiviral treatment when appropriate. Our literature review revealed that a majority of relevant studies found an epidemiological association between HCV infection and WM but that this conclusion was not unanimous.

The literature for treatment of such patients is less robust. Available data, limited to case reports, suggest that initial antiviral treatment of the HCV may be a viable option. However, further investigation is needed to compare this approach systematically with initial treatment of WM, cotreatment of both conditions, and other treatment strategies.

## Figures and Tables

**Table 1:** Publications reviewed. Second column includes whether the authors concluded that there is an association between hepatitis C virus infection and Waldenström macroglobulinemia in their cohort and a brief summary of relevant findings. Third column summarizes the treatment strategy for patients with comorbid hepatitis C virus infection and Waldenström macroglobulinemia.

Article	Did authors conclude an association between HCV and WM?	Treatment given for HCV and WM, and patient outcomes
Santini et al. 1993 [6]	Yes. 6/6 WM patients were HCV positive by viral PCR.	n/a

Mussini et al. 1995 [21]	Yes, in setting of cryoglobulinemia. Of WM cases with cryoglobulins, 2/3 were positive for HCV RNA. 0/12 WM cases without cryoglobulins were positive for HCV.	n/a

Andreone et al. 1995 [22]	No. Hypothesize that association between HCV and NHL may be due by confounding factor of transfusions.	n/a

Custodi et al. 1995 [13]	No. 0/6 HCV positive in 6 cases of familial occurrence of IgM-k gammopathy.	n/a

Izumi et al. 1996 [23]	Unlikely. 1/4 patients with WM were HCV positive.	n/a

Izumi et al. 1996 [20]	Case report: 55-year-old man with HCV, WM, and hepatocellular carcinoma.	Melphalan and prednisolone + vincristine. Death from liver failure 5 years after diagnosis.

Silvestri et al. 1996 [24]	Yes, in setting of cryoglobulinemia. 1/20 WM patients with cryoglobulinemia were HCV positive. 0/19 WM patients without cryoglobulinemia were HCV positive.	n/a

Silvestri et al. 1996 [25]	Yes. 30% of patients with immunocytoma were HCV positive.	n/a

Silvestri et al. 1997 [10]	Yes. Of the HCV-positive, cryoglobulin-producing NHL cases, immunocytoma was most frequent (16/21).	n/a

Izumi et al. 1997 [9]	Yes. 1/4 patients with WM were HCV positive.	n/a

Silvestri and Baccarani 1997 [15]	Yes. 26–49% of lymphoplasmacytoid lymphomas were HCV positive.	n/a

Zignego et al. 1997 [26]	Yes. Mechanism of HCV infection leading to B-cell NHL may be through vasculitis and triggering of lymphoproliferative disorder.	n/a

Silvestri et al. 1998 [27]	Yes. 18/70 WM cases were HCV positive.	10 WM patients treated with IFN alpha for 6 to 12 months. Four patients were resistant and received fludarabine without response.

Trotter 1999 [19]	Case report: 45-year-old man with cirrhosis and HCV-induced cirrhosis and WM.	Recurrent plasmapheresis resolved symptoms.

Ahmed et al. 1999 [28]	Yes. 3/3 patients with WM tested for HCV were positive.	n/a

Vallisa et al. 1999 [4]	Yes. Prevalence of HCV infection among patients with NHL was 37.1%.	n/a

Silvestri et al. 2000 [5]	Yes. 26% to 49% of cases of WM were HCV positive.	n/a

Dammacco et al. 2000 [29]	Yes. HCV reported to be lymphotropic and may trigger clonal B-cell proliferation, leading to the progression to lymphoid malignancy.	n/a

Rabkin et al. 2002 [12]	No. 4/95 lymphoma pts had HCV EIAs but none confirmed by recombinant immunoblot assay.	n/a

Musto 2002 [30]	Yes. HCV reported to be lymphotropic and may also trigger clonal B-cell proliferation, leading to malignancy.	n/a

Álvarez-Ruiz et al. 2004 [17]	Case report: 50-year-old man with HCV and mixed cryoglobulinemia associated with WM.	IFN alpha and ribavirin for 9 months. HCV became undetectable and BM biopsy showed no lymphoid infiltration. Liver transplant performed.

Veneri et al. 2004 [11]	No. HCV prevalence in WM patients similar to normal population.	n/a

Neri et al. 2005 [18]	Case report: 63-year-old woman with WM and type I cryoglobulinemia.	Pef IFN alpha-2b treatment for 6 months. Symptoms returned after IFN treatment. Resolution of presenting skin symptoms after cyclophosphamide and prednisone addition.

Leleu et al. 2007 [2]	No. 0 out of 100 WM patients studied was HCV positive.	n/a

Giordano et al. 2007 [7]	Yes. HCV infection with increased risk of WM, HR of 2.76 (95% CI, 2.01–3.79).	n/a

Schöllkopf et al. 2008 [8]	Yes. OR 5.2 (1.0–26.4) of HCV positivity in WM.	n/a

Nicolosi Guidicelli et al. 2012 [3]	No. Only geographic differences in prevalence of HCV in NHL.	n/a

Arcaini et al. 2012 [16]	Yes. Lymphoma regression following IFN-based treatment.	Interferon +/− ribavirin effective in HCV + patients with indolent lymphoma.

## Abbreviations

WM: Waldenström macroglobulinemia
Ig: Immunoglobulin
RR: Relative risk
OR: Odds ratio
HCV: Hepatitis C virus
NHL: Non-Hodgkin lymphoma
IFN: Interferon.

## References

[1] Agnello V, Chung RT, Kaplan LM (1992). A role for hepatitis C virus infection in Type II cryoglobulinemia. *The New England Journal of Medicine*.

[2] Leleu X, O'Connor K, Ho AW (2007). Hepatitis C viral infection is not associated with Waldenstrom's Macroglobulinemia. *The American Journal of Hematology*.

[3] Nicolosi Guidicelli S, Lopez-Guillermo A, Falcone U (2012). Hepatitis C virus and GBV-C virus prevalence among patients with B-cell lymphoma in different European regions: a case-control study of the International Extranodal Lymphoma Study Group. *Hematological Oncology*.

[4] Vallisa D, Bertè R, Rocca A (1999). Association between hepatitis C virus and non-Hodgkin's lymphoma, and effects of viral infection on histologic subtype and clinical course. *American Journal of Medicine*.

[5] Silvestri F, Sperotto A, Fanin R (2000). Hepatitis c and lymphoma. *Current Oncology Reports*.

[6] Santini GF, Crovatto M, Modolo ML (1993). Waldenstrom macroglobulinemia: a role of HCV infection?. *Blood*.

[7] Giordano TP, Henderson L, Landgren O (2007). Risk of non-Hodgkin lymphoma and lymphoproliferative precursor diseases in US veterans with hepatitis C virus. *The Journal of the American Medical Association*.

[8] Schöllkopf C, Smedby KE, Hjalgrim H (2008). Hepatitis C infection and risk of malignant lymphoma. *International Journal of Cancer*.

[9] Izumi T, Sasaki R, Tsunoda S, Akutsu M, Okamoto H, Miura Y (1997). B cell malignancy and hepatitis C virus infection. *Leukemia*.

[10] Silvestri F, Barillari G, Fanin R (1997). Hepatitis C virus infection among cryoglobulinemic and non- cryoglobulinemic B-cell non-Hodgkin's lymphomas. *Haematologica*.

[11] Veneri D, Aqel H, Franchini M, Meneghini V, Krampera M (2004). Prevalence of hepatitis C virus infection in IgM-type monoclonal gammopathy of uncertain significance and Waldenström macroglobulinemia. *American Journal of Hematology*.

[12] Rabkin CS, Tess BH, Christianson RE (2002). Prospective study of hepatitis C viral infection as a risk factor for subsequent B-cell neoplasia. *Blood*.

[13] Custodi P, Cerutti A, Cassani P, Perazzi C, Ravanini P, Fortina G (1995). Familial occurrence of IgMk gammapathy: no involvement of HCV infection.. *Haematologica*.

[14] Ghobrial IM (2013). Choice of therapy for patients with Waldenstrom macroglobulinemia. *Journal of Clinical Oncology*.

[15] Silvestri F, Baccarani M (1997). Hepatitis C virus-related lymphomas. *British Journal of Haematology*.

[16] Arcaini L, Merli M, Volpetti S, Rattotti S, Gotti M, Zaja F (2012). Indolent B-Cell lymphomas associated with HCV infection: clinical and virological features and role of antiviral therapy. *Clinical and Developmental Immunology*.

[17] Álvarez-Ruiz SB, García-Río I, Aragüés M (2004). Leucocytoclastic vasculitis, hepatitis C virus-associated mixed cryoglobulinaemia with biclonal gammopathy and Waldenström macroglobulinaemia. *British Journal of Dermatology*.

[18] Neri S, Pulvirenti D, Mauceri B, Ignaccolo L, Azzolina R (2005). A case of progression from type II cryoglobulinaemia to Waldenstrom's macroglobulinaemia in a patient with chronic hepatitis C. *Clinical and Experimental Medicine*.

[19] Trotter JF (1999). Waldenstrom's macroglobulinemia in a patient with cirrhosis and chronic hepatitis C infection. *The American Journal of Medicine*.

[20] Izumi T, Sasaki R, Tsunoda S (1996). Sequential occurrence of hepatocellular carcinoma following Waldenström's macroglobulinemia: the pathogenetic role of chronic liver disease. *Internal Medicine*.

[21] Mussini C, Ghini M, Mascia MT (1995). Monoclonal gammopathies and hepatitis C virus infection. *Blood*.

[22] Andreone P, Gramenzi A, Cursaro C, Bernardi M (1995). Hepatitis C virus infection and lymphoproliferative disorders. *Blood*.

[23] Izumi T, Sasaki R, Shimizu R (1996). Hepatitis C virus infection in Waldenström's macroglobulinemia. *American Journal of Hematology*.

[24] Silvestri F, Barillari G, Fanin R (1996). Risk of hepatitis C virus infection, Waldenstrom's macroglobulinemia, and monoclonal gammopathies. *Blood*.

[25] Silvestri F, Pipan C, Barillari G (1996). Prevalence of hepatitis C virus infection in patients with lymphoproliferative disorders. *Blood*.

[26] Zignego AL, Ferri C, Giannini C (1997). Hepatitis C virus infection in mixed cryoglobulinemia and B-cell Non-Hodgkin's lymphoma: evidence for a pathogenetic role. *Archives of Virology*.

[27] Silvestri F, Barillari G, Fanin R (1998). Impact of hepatitis C virus infection on clinical features, quality of life and survival of patients with lymphoplasmacytoid lymphoma/immunocytoma. *Annals of Oncology*.

[28] Ahmed S, Shurafa MS, Bishop CR, Varterasian M (1999). Waldenström’s macroglobulinemia in young African-American adults. *The American Journal of Hematology*.

[29] Dammacco F, Sansonno D, Piccoli C, Racanelli V, D'Amore FP, Lauletta G (2000). The lymphoid system in hepatitis C virus infection: Autoimmunity, mixed cryoglobulinemia, and overt B-cell malignancy. *Seminars in Liver Disease*.

[30] Musto P (2002). Hepatitis C virus infection and B-cell non-Hodgkin's lymphomas: more than a simple association. *Clinical Lymphoma*.

